# SV2 Mediates Entry of Tetanus Neurotoxin into Central Neurons

**DOI:** 10.1371/journal.ppat.1001207

**Published:** 2010-11-24

**Authors:** Felix L. Yeh, Min Dong, Jun Yao, William H. Tepp, Guangyun Lin, Eric A. Johnson, Edwin R. Chapman

**Affiliations:** 1 Department of Physiology, Howard Hughes Medical Institute, University of Wisconsin, Madison, Wisconsin, United States of America; 2 New England Primate Research Center, Department of Microbiology and Molecular Genetics, Harvard Medical School, Southborough, Massachusetts, United States of America; 3 Department of Bacteriology, University of Wisconsin, Madison, Wisconsin, United States of America; The University of Texas-Houston Medical School, United States of America

## Abstract

Tetanus neurotoxin causes the disease tetanus, which is characterized by rigid paralysis. The toxin acts by inhibiting the release of neurotransmitters from inhibitory neurons in the spinal cord that innervate motor neurons and is unique among the clostridial neurotoxins due to its ability to shuttle from the periphery to the central nervous system. Tetanus neurotoxin is thought to interact with a high affinity receptor complex that is composed of lipid and protein components; however, the identity of the protein receptor remains elusive. In the current study, we demonstrate that toxin binding, to dissociated hippocampal and spinal cord neurons, is greatly enhanced by driving synaptic vesicle exocytosis. Moreover, tetanus neurotoxin entry and subsequent cleavage of synaptobrevin II, the substrate for this toxin, was also dependent on synaptic vesicle recycling. Next, we identified the potential synaptic vesicle binding protein for the toxin and found that it corresponded to SV2; tetanus neurotoxin was unable to cleave synaptobrevin II in SV2 knockout neurons. Toxin entry into knockout neurons was rescued by infecting with viruses that express SV2A or SV2B. Tetanus toxin elicited the hyper excitability in dissociated spinal cord neurons - due to preferential loss of inhibitory transmission - that is characteristic of the disease. Surprisingly, in dissociated cortical cultures, low concentrations of the toxin preferentially acted on excitatory neurons. Further examination of the distribution of SV2A and SV2B in both spinal cord and cortical neurons revealed that SV2B is to a large extent localized to excitatory terminals, while SV2A is localized to inhibitory terminals. Therefore, the distinct effects of tetanus toxin on cortical and spinal cord neurons are not due to differential expression of SV2 isoforms. In summary, the findings reported here indicate that SV2A and SV2B mediate binding and entry of tetanus neurotoxin into central neurons.

## Author Summary

Tetanus neurotoxin is one of the most deadly bacterial toxins known and is the causative agent for the disease tetanus, also known as lockjaw. Tetanus neurotoxin utilizes motor neurons as a means of transport in order to enter the spinal cord. Once in the spinal cord, the toxin leaves motor neurons and enters inhibitory neurons through a “Trojan-horse” strategy, thereby preventing the release of inhibitory neurotransmitters onto motor neurons. This causes hyper-excitability of the motor neuron and excessive release of acetylcholine at the neuromuscular junction, resulting in rigid paralysis. There is a major gap in our understanding of the mechanism by which tetanus neurotoxin enters neurons. In the current study we discovered that the “Trojan-horse”, utilized by tetanus neurotoxin to enter central neurons, corresponds to recycling synaptic vesicles. Furthermore, we discovered that SV2 is critical for the binding and entry of tetanus neurotoxin into these neurons. These findings will enable further development of drugs that antagonize the action of the toxin and will also aid in the development of drug delivery systems that target spinal cord neurons.

## Introduction

The *Clostridium* genus of bacteria are responsible for the production of the clostridial neurotoxins (CNTs), which include both tetanus neurotoxin (TeNT) and seven botulinum neurotoxins (BoNT/A–G) [1]. TeNT is synthesized by *Clostridium tetani*, and is one of the most toxic substances known to humans; it causes the disease tetanus [2], [3]. Spores enter via deep wounds where they germinate in the anaerobic environment, releasing TeNT via autolysis [1]. Upon exposure to fatal levels of the toxin, patients eventually die of respiratory or heart failure, thereby generating a rich anaerobic environment in which the bacteria can proliferate [4]. Tetanus kills hundreds of thousands of people each year in countries in which regular tetanus vaccinations are not carried out [1].

Structurally, the CNTs are 150 kDa proteins composed of a heavy chain (HC) and a light chain (LC) that are linked through a disulfide bond. The 100 kDa HC, which has two functional domains, mediates binding to neuronal receptors and also creates a pore that mediates the translocation of the 50 kDa LC, a zinc-dependent endoprotease, into the cytosol [1], [5]. The LC then cleaves one or more of three soluble N-ethylmaleimide-sensitive fusion protein receptor (SNARE) proteins: BoNT/A and E cleave the plasma membrane protein SNAP-25 (synaptosomal-associated protein of 25 kDa); BoNT/B, D, F, G and TeNT cleave the vesicle protein synaptobrevin (syb); and BoNT/C cleaves both SNAP-25 and syntaxin-1 [6], [7], [8], [9], [10]. Assembly of syb•syntaxin•SNAP-25 into parallel four-helix bundles is thought to pull the vesicle and plasma membranes together to drive membrane fusion [11]. Cleavage of these SNAREs by the CNTs either severs them from the membrane or disrupts their ability to assemble into stable/functional fusion complexes, thereby blocking synaptic vesicle (SVs) exocytosis and neurotransmitter release [1], [12].

While TeNT causes rigid paralysis, the BoNTs cause flaccid paralysis [13]. These opposite symptoms are the result of different sites of action. The BoNTs exert their effects at the neuromuscular junction (NMJ) by cleaving one or more of the three synaptic SNARE proteins. While TeNT also enters the nervous system via presynaptic terminals of the α-motor neuron (MN) at the NMJ, it does not act at this site but rather undergoes retrograde transport into the spinal cord. To achieve this, TeNT localizes to lipid rafts that contain high local concentrations of cholesterol, polysialogangliosides (PSGs), and glycophospoinositol (GPI)-anchored proteins in the terminals of MNs [14], [15]. Once bound, TeNT is internalized into non-acidified vesicles that harbor growth factor receptors [16]. The TeNT-harboring vesicle is sorted via a Rab 5/7 dependent pathway and transported back to the cell body of the MN [17]. TeNT then undergoes transcytosis by being released from the MNs such that it enters upstream inhibitory neurons to cleave SNAREs and inhibit transmitter release [18], [19].

The pathway by which TeNT acts on inhibitory neurons occurs via four steps. 1) TeNT binds to presynaptic terminals through interactions with a “dual receptor” composed of lipid and protein components that, together, form high-affinity receptors [20]. As opposed to the other CNTs, which harbor one PSG binding site (which is the lipid component of the dual receptor), it was discovered that TeNT contains two binding sites for PSGs. Among the PSGs, TeNT exhibits stronger interactions with GT1b, GD1b, and GQ1b [1]. Furthermore, mice lacking PSGs were resistant to TeNT as compared to wild-type (WT) mice [21], [22]. Experiments performed with spinal cord neurons and rat brain membranes also indicated the presence of a protease-sensitive protein receptor; however, the identity of this protein remains unknown [23], [24], [25]. 2) Once bound to the membrane, TeNT is internalized via endocytosis. 3) Following endocytosis, acidification of the vesicle lumen triggers conformational changes in the HC which cause it to form a translocation channel or pore in the vesicular membrane. The LC translocates through the HC pore and the disulfide bond connecting the HC and the LC becomes reduced in the cytosol. 4) The LC cleaves syb to inhibit SV exocytosis. The resultant loss of inhibitory neurotransmission results in hyper-excitability of the MN, thereby enhancing release of acetylcholine and producing rigid paralysis [26].

The protein receptors for BoNT/A, B ,E and G have recently been identified [27], [28], [29], [30], [31], [32], [33]. The unique ability of TeNT to shuttle from the periphery to the central nervous system has made determining the receptor(s) for this toxin a greater challenge. At present, the route of entry of TeNT into central neurons is unclear, with some reports indicating that, in hippocampal cultures, binding and entry was dependent on SV recycling [34]. However, other studies indicate that entry of TeNT into spinal cord neurons was mediated by non-SV carriers [35], [36], and thus this question remains an open issue. Furthermore, the receptor-binding domain of TeNT was reported to bind to a GPI-anchored protein that was sensitive to phosphoinositol specific phosholipase C (PI-PLC) treatment in MNs, spinal cord neurons, and PC12 cells (rat pheochromocytoma cell line). This putative receptor was identified as Thy-1 [35], [37], but whether Thy-1 is required for binding and uptake of TeNT has yet to be tested.

As previously hypothesized, the protein receptors for TeNT in inhibitory neurons and MNs are most likely to be distinct proteins [1]. Namely, the receptor that is present in MNs directs TeNT into a non-acidified compartment to circumvent translocation, whereas the receptor that is expressed by inhibitory neurons is targeted to vesicular structures that undergo acidification, thus allowing for translocation [16]. Secondly, we note that dogs and cats are orders of magnitude more resistant to TeNT when injected into the periphery as compared to into the spinal cord [1], [38], [39]. It is therefore possible that dogs and cats have substantial sequence variation in the MN receptor that prevents proper interactions with TeNT, resulting in reduced uptake and retrograde transport.

The identification of the somatic and central neuron protein receptors for TeNT will aid in developing antagonists to combat the disease, especially in nations without a tetanus vaccination regimen [1]. Due to the toxin's unique ability to be transported from MNs into inhibitory interneurons in the central nervous system, the findings described here will also aid in the development of novel drug delivery methods for the treatment of central nervous system diseases [40].

## Results

### TeNT binds to central neurons via recycling synaptic vesicles

As alluded to before, there are conflicting reports regarding the mode of entry for TeNT into central neurons. It was first suggested that in hippocampal neurons, TeNT enters through recycling SVs [34]. However, a later paper reported that the sensitivity of mouse cerebellar slices to TeNT was reduced upon treatment with PI-PLC, indicating the receptor had a GPI-anchor moiety [35]. Furthermore in another study, gold-labeled TeNT was not commonly found in SVs in cultured spinal cord neurons [36]. These results argued against a SV uptake pathway because these vesicles are devoid of GPI-linked proteins [41]. To address these apparent disparities, we utilized a recombinant fragment of TeNT, corresponding to its receptor-binding domain, to carry out binding assays using cultured neurons. This fragment harbored a 3x-FLAG epitope tag that was used for our imaging studies [37], [42], [43]. This C-terminal subdomain of the heavy chain (HCR/T) has been shown to have the same binding, uptake, and retrograde transport properties as the full length and enzymatically active holotoxin [44], [45], [46], [47]

**Figure 1:**
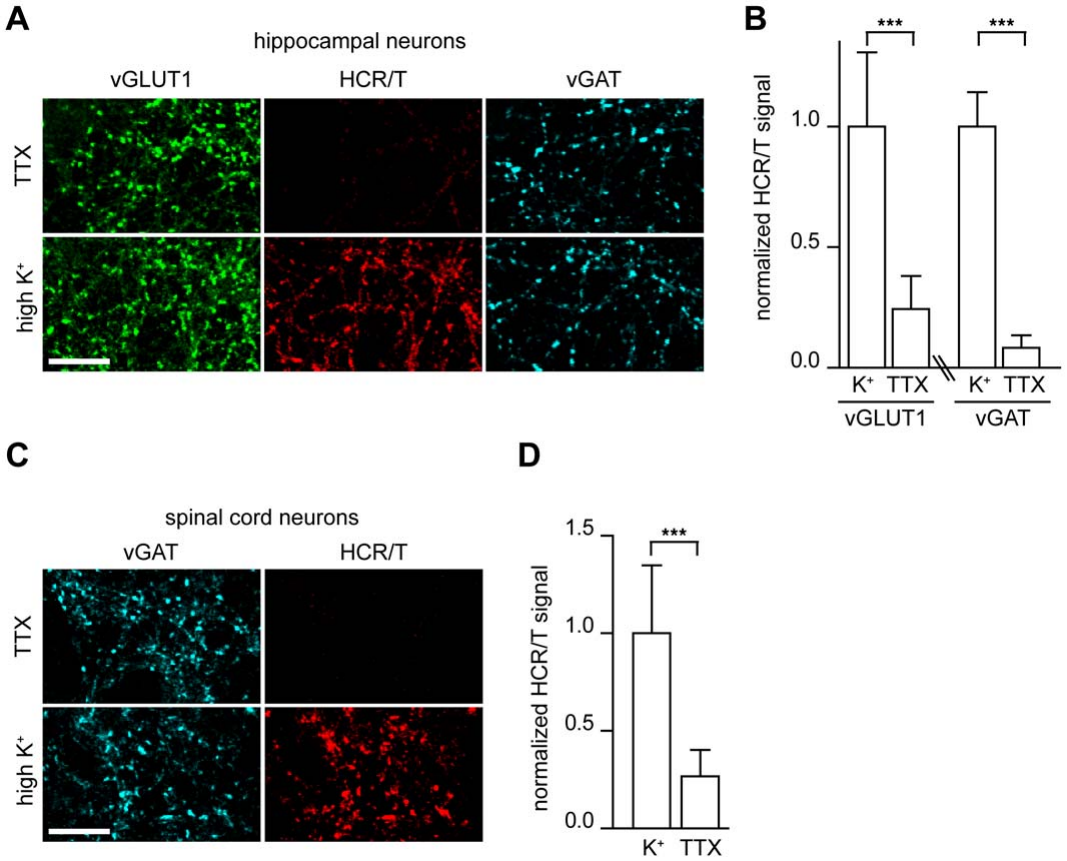
TeNT binds to central neurons via recycling synaptic vesicles. (A) Cultured rat hippocampal neurons were incubated with the receptor-binding domain of TeNT (HCR/T, 50 nM) either in non-depolarizing conditions (TTX: 5 mM KCl, 0 mM Ca^2+^, 1 µM TTX) or depolarizing conditions (high K^+^ buffer: 55 mM KCl, 2 mM Ca^2+^). Cells were processed for immunocytochemistry and HCR/T was detected using a mouse anti-FLAG tag monoclonal antibody. Vesicular glutamate transporter 1 (vGLUT1) antibodies were used to mark excitatory synapses and antibodies against vesicular GABA transporter (vGAT) were used to mark inhibitory terminals. The binding of HCR/T to neurons was markedly enhanced under depolarizing conditions. Scale bars in all figures are 10 µm unless otherwise indicated. (B) Quantification of HCR/T staining intensity at excitatory/inhibitory terminals normalized to VGLUT1 or vGAT intensity. Error bars represent SD, n = 9, ***p≤0.001. (C) Dissociated spinal cord neurons were incubated with HCR/T (50 nM) in TTX buffer or high K+ buffer. Binding of HCR/T was markedly increased under depolarizing conditions. (D) Quantification of HCR/T to vGAT staining intensity ratio. Error bars represent SD, n = 18, ***p≤0.001.

Extracellular exposure of neurons to solutions that contain high concentrations of potassium results in membrane depolarization; calcium then enters pre-synaptic terminals through voltage-gated calcium channels thereby triggering SV exocytosis. We applied this technique to investigate the binding of HCR/T to hippocampal neurons under depolarizing and non-depolarizing conditions. We utilized hippocampal neurons as they have proven to be a useful model system that has been pivotal in the identification of CNT receptors [27], [28], [30], [34]. Incubation of HCR/T with these neurons in a buffer containing tetrodotoxin (TTX), which blocks action potentials and as a result active SV recycling, resulted in little binding of HCR/T. However, when experiments were performed in the presence of high potassium buffer, a large increase in HCR/T fluorescence was observed (Figure 1A). Quantitative analysis revealed a 3-fold increase in the fluorescence intensity of HCR/T at excitatory terminals (vGLUT1) and a 10-fold increase at inhibitory terminals (vGAT) upon stimulation with high potassium (Figure 1B). These results suggest that the binding partner for TeNT is localized to the lumen of SVs.

TeNT causes disease by exerting its effects at inhibitory neurons in the spinal cord, so we extended our experiments to cultured dissociated spinal cord neurons obtained from embryonic rats. Again, HCR/T was incubated in TTX (non-depolarizing) and high potassium (depolarizing) conditions, and we observed a 4-fold increase of HCR/T binding upon depolarization of inhibitory boutons (Figure 1C–D). The increase in TeNT binding to spinal cord terminals under high potassium versus TTX conditions strongly suggests that the binding partner for TeNT is localized to SVs.

### Cleavage of synaptobrevin II is dependent on synaptic vesicle recycling

**Figure 2:**
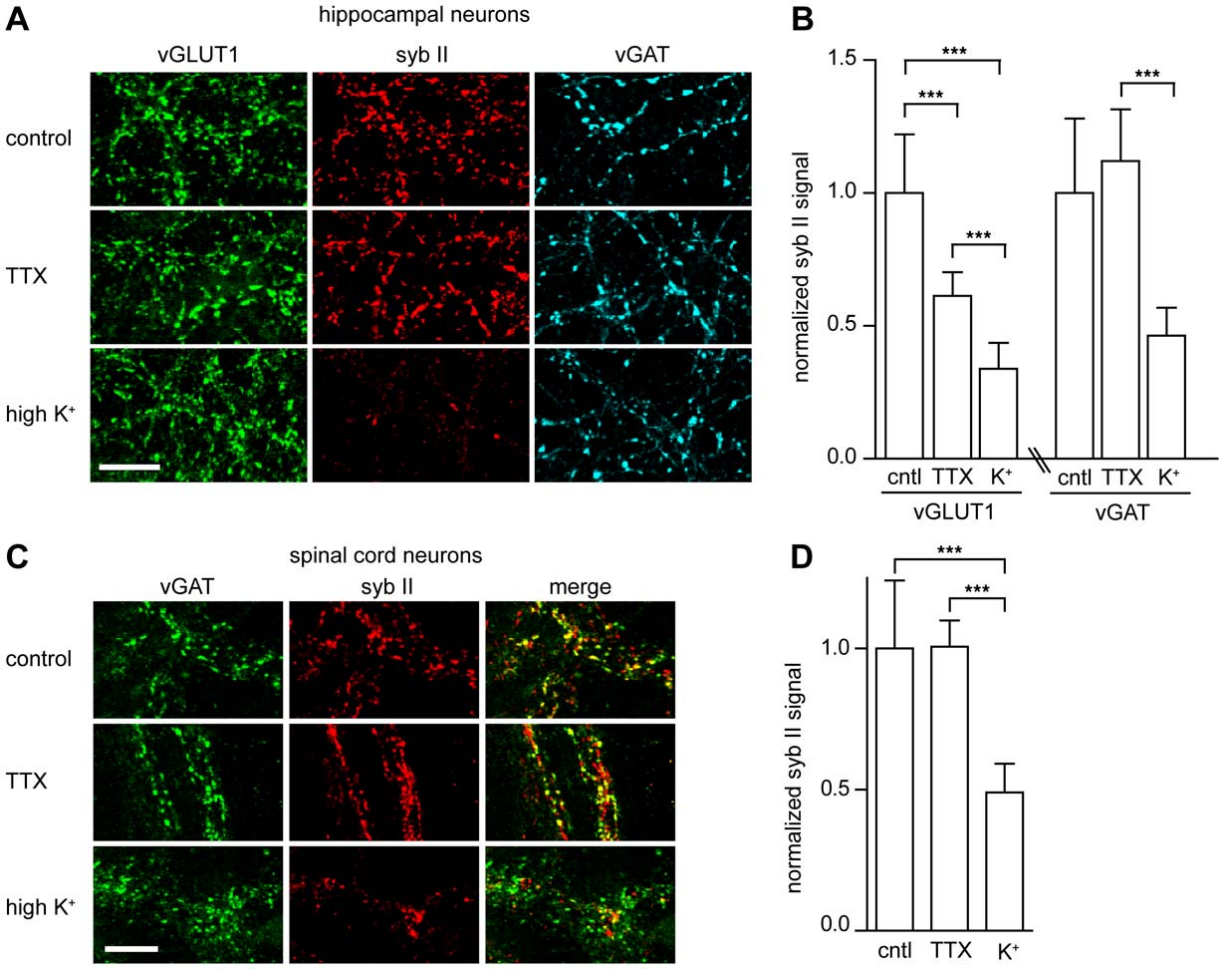
TeNT requires actively recycling synaptic vesicles to enter neurons and cleave synaptobrevin II. (A) Cultured hippocampal neurons were incubated with full-length TeNT (5 nM) in non-depolarizing (TTX) or depolarizing (high K+) buffer for 3 minutes, washed, then returned to media and incubator for 4 hours. Cells were processed for immunostaining; the synaptobrevin II (syb II, Cl 69.1) signal was significantly reduced under depolarizing conditions. (B) Quantification of syb II intensity at excitatory or inhibitory terminals normalized to VGLUT1 or vGAT intensities, respectively. Error bars represent SD, n = 9, ***p≤0.001. (C) Cultured spinal cord neurons were incubated with TeNT holotoxin (500 pM) in TTX or high K+ buffers for 3 minutes, washed, then returned to media and incubated for 4 hours. (D) Quantification of syb II to vGAT fluorescence intensity ratios. Error bars represent SD, n = 9, ***p≤0.001.

The previous experiments strongly suggest that TeNT binds to a receptor that is a resident of SVs, so we next determined whether this interaction results in functional entry of the toxin. To test this idea, we determined whether the ability of TeNT holotoxin to enter neurons and cleave syb II also depended on SV recycling. We employed a monoclonal antibody raised against syb II (Cl. 69.1) that cannot recognize the enzymatically cleaved form of the protein. We began by comparing the ability of TeNT to cleave syb II under TTX (which blocks action potentials to inhibit SV recycling) or high potassium (which depolarizes neurons to drive SV recycling) conditions. We observed that syb II fluorescence was markedly reduced when hippocampal neurons were depolarized with potassium to drive SV recycling (Figure 2A); this occurred at both excitatory and inhibitory nerve terminals (Figure 2B). Syb II immunofluorescence was reduced in excitatory terminals by 39% in the TTX condition and was further reduced by 65% of under depolarizing conditions, as compared to control. Inhibitory terminals exhibited no significant reduction of syb II immunofluorescence under TTX conditions, but a 53% reduction was observed in neurons that had been depolarized (Figure 2B).

To test the physiological relevance of these observations, we monitored syb II levels in spinal cord neurons (Figure 2C) and observed that under high potassium conditions syb II immunofluorescence was reduced by 50% as compared to control and non-stimulating (i.e. TTX) conditions (Figure 2D). These data further reinforce the idea that TeNT entry into inhibitory spinal cord neurons occurs through recycling SVs, relying on a receptor that is localized to SVs.

### Entry of TeNT into central neurons is abrogated by inhibitors of exocytosis/endocytosis

**Figure 3:**
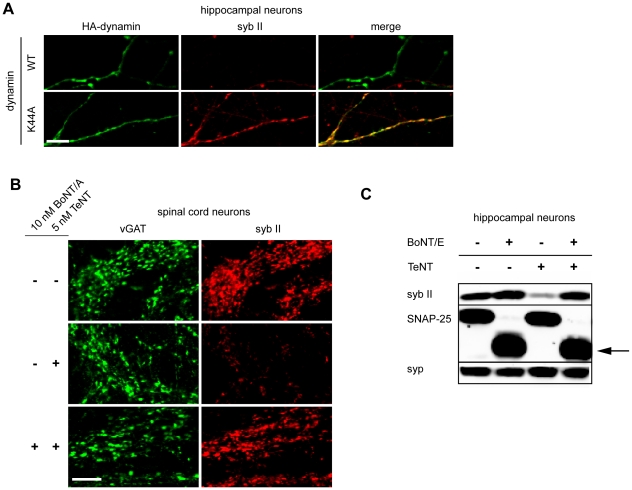
Inhibition of exocytosis or endocytosis prevents the entry of TeNT into neurons. (A) Hippocampal neurons were transfected with HA tagged wild type or K44A dynamin 1. Syb II was protected from cleavage by TeNT in hippocampal neurons expressing the dominant negative form of dynamin as compared to control neurons. (B) Spinal cord neurons that were pretreated with 10 nM BoNT/A, to inhibit exocytosis, failed to take-up TeNT (5 nM) at inhibitory boutons. (C) Hippocampal neurons were treated with TeNT (5 nM) which had or had not been pretreated with BoNT/E (500 pM). Cell lysates were loaded and subjected to SDS-PAGE and immunoblot analysis. Pretreatment with BoNT/E prevented entry of TeNT and cleavage of syb II. The arrow indicates the BoNT/E cleaved form of SNAP-25. Synaptophysin (syp), another SV protein, served as a loading control.

To further confirm the requirement of SVs for the entry of TeNT into neurons, we carried out experiments in which we inhibited steps in the SV cycle and assayed for protection from the toxin. For these experiments we took advantage of a dynamin point mutant that interferes with the GTPase function of the protein. This mutant (K44A) acts as a dominant negative that inhibits endocytosis [48]. As shown in Figure 3A, neurons that expressed the K44A mutant were protected from the entry of TeNT, as evidenced by the lack of cleavage of syb II, as compared to neurons that expressed WT dynamin.

We next determined whether we could prevent TeNT entry by inhibiting the exocytosis (and thereby blocking compensatory endocytosis) of SVs. As BoNT/A and BoNT/E cleave SNAP-25 instead of syb II, we were able to inhibit exocytosis in neurons while still monitor the entry of TeNT through cleavage of syb II. Hence, we first pre-treated spinal cord neurons with BoNT/A to cleave SNAP-25 and inhibit exocytosis [1], and then we subsequently assayed for entry of TeNT by monitoring cleavage of syb II. We observed that syb II was largely protected from the effects of TeNT at inhibitory terminals when exocytosis had been inhibited by prior treatment with BoNT/A (Figure 3B). Similar results were observed using hippocampal neurons that had been pretreated with BoNT/E, which also cleaves SNAP-25 (Figure 3C). Together, the results reported thus far establish the notion that the primary route for TeNT-induced toxicity is through recycling SVs and not through an alternative pathway.

### SV2A/B mediate binding and entry of TeNT

**Figure 4:**
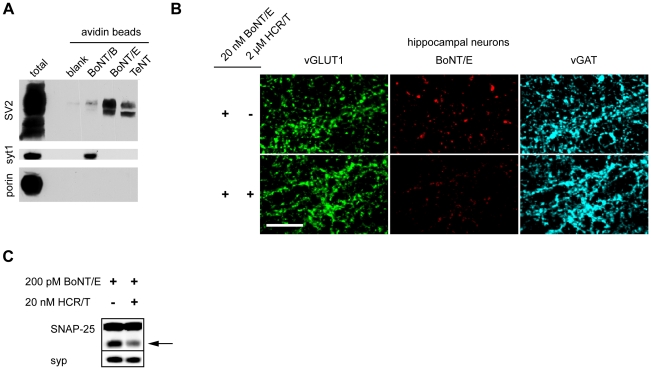
TeNT associates with SV2. (A) Biotinylated BoNT/B, E and TeNT were pre-incubated with neutravidin beads and then mixed with brain detergent extract. Beads were washed and bound material was subjected to SDS-PAGE and immunoblot analysis. BoNT/B and BoNT/E both associated with their receptors, synaptotagmin 1 (syt1, Cl 69.1) and SV2 (pan-SV2), respectively. TeNT showed a strong association with SV2, but not with other synaptic vesicle proteins such as syt1 and synaptoporin (porin). (B) BoNT/E (20 nM) was analyzed for binding to hippocampal neurons in the presence and absence of 2 µM HCR/T. Binding of BoNT/E to neurons was substantially reduced by HCR/T, indicating competition for binding to SV2. (C) Hippocampal neurons were treated with 200 pM BoNT/E in the presence and absence of 20 nM HCR/T. The arrow indicates the BoNT/E cleaved form of SNAP-25. Immunoblot analysis revealed that the presence of HCR/T resulted in the reduced cleavage of SNAP-25 by BoNT/E, again indicating competition for binding to the same receptor.

To identify the potential SV binding partner for TeNT, we biotinylated this toxin, as well as BoNT/B, BoNT/E (as controls), and bound them to neutravidin beads, through biotin-avidin interactions. After incubating the toxin-linked beads with brain detergent extracts (BDE), we screened for bound SV proteins. Previously it has been shown that the receptor for BoNT/B is synaptotagmin (syt) I/II and the receptor for BoNT/E is SV2A/B [29], [30]. Consistent with previous reports, we detected that BoNT/B associated with syt I and BoNT/E with SV2, but surprisingly, we observed that TeNT also strongly associated with SV2 (Figure 4A). To further confirm that TeNT associated with SV2, we used HCR/T to see if it could compete with BoNT/E for binding to its native receptor, SV2, on hippocampal neurons. A 100-fold molar excess of HCR/T, compared to BoNT/E, markedly reduced BoNT/E binding to nerve terminals in this preparation (Figure 4B). Moreover, competition of HCR/T with BoNT/E in hippocampal neurons reduced the extent of cleavage of SNAP-25 (Figure 4C). These data are in agreement with previous studies on the NMJ that also demonstrate HCR/T can antagonize BoNT/E entry [49], [50]. The ability of the receptor-binding domain of TeNT to reduce BoNT/E binding and entry into hippocampal neurons - through competition for binding SV2 - further supports the idea that TeNT utilizes SV2 as its receptor protein.

**Figure 5:**
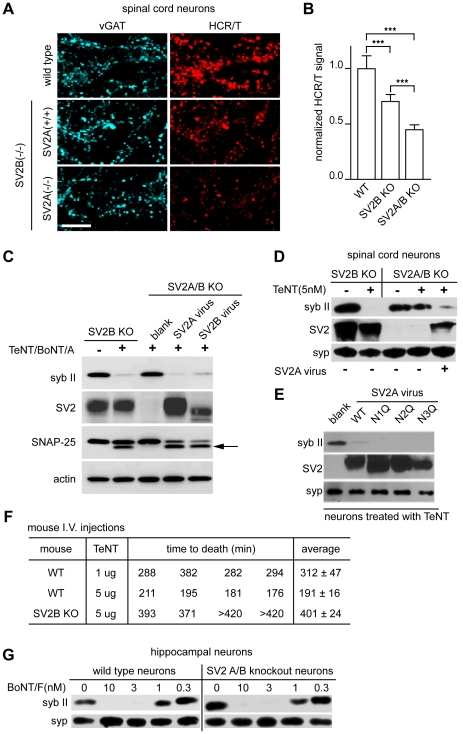
SV2A/B KO neurons are resistant to TeNT. (A) Mouse spinal cord neurons with the following genotypes: WT, SV2B KO [SV2A (+/+) SV2B (−/−)], and SV2A/B KO [SV2A (−/−) SV2B (−/−)], were exposed to HCR/T (50 nM). HCR/T fluorescence in SV2A/B KO neurons was dramatically reduced as compared to WT and SV2B KO. (B) Quantification of HCR/T binding: fluorescence was reduced by 30% and 50% for SV2B KO and SV2A/B KO neurons, respectively. Error bars represent SD, WT n = 9, SV2B KO n = 11, SV2A/B KO n = 12, ***p≤0.001. (C) SV2B KO and SV2A/B KO cultures were exposed to TeNT (20 nM) and BoNT/A (10 nM). Cell lysates were subjected to immunoblot analysis and probed for syb II, SV2, SNAP-25, and actin. Syb II in SV2A/B KO neurons was largely protected from TeNT action until resensitized through lentiviral expression of SV2A or SV2B; arrow indicates the BoNT/A cleaved form of SNAP-25. (D) SV2B KO and SV2A/B KO spinal cord neurons from were assayed for susceptibility to TeNT. Syb II was cleaved by 5 nM TeNT in SV2B KO neurons, while syb II was protected from TeNT in SV2A/B KO neurons. SV2A/B KO neurons could be resensitized to TeNT, upon lentiviral expression of SV2A. (E) Three putative glycosylation sites in SV2A were removed by creating N to Q mutants (residues 498, 548 and 573) and were expressed in SV2A/B KO neurons along with WT SV2A. Syb II was cleaved by TeNT in neurons reinfected with WT SV2A as well as the three mutants. (F) WT and SV2B KO mice were injected with the indicated amounts of TeNT and their time-to-death was recorded. SV2B KO mice were more than five-times more resistant to TeNT as compared to their WT counterparts. (G) WT and SV2A/B KO neurons were cultured and treated with BoNT/F at the indicated concentrations. Cell lysates were probed for syb II and syp by immunoblot analysis. WT and SV2A/B KO neurons exhibited similar sensitivities to BoNT/F.

SV2 is a 12 transmembrane protein that is heavily glycosylated and is homologous to transmembrane transporters. SV2 exists in 3 isoforms - A, B, and C - and SV2 A and B, but not C, knock-out (KO) mice have been generated [51], [52]. We utilized dissociated spinal cord neurons from SV2 KO mice in order to directly determine whether SV2 was critical for the action of TeNT at a functional level. We observed significant reductions of HCR/T binding to inhibitory terminals in KO mice (Figure 5A). Compared to WT, there was a 30% reduction in HCR/T binding to SV2B KO spinal cord neurons and a 55% reduction of HCR/T binding to inhibitory terminals of SV2A/B KO neurons (Figure 5B). These results further demonstrate that SV2 plays a critical role in the binding of TeNT to inhibitory terminals in the spinal cord.

To investigate whether SV2 was necessary for entry of TeNT, we cultured SV2A/B KO hippocampal neurons which express little SV2C [27] and examined whether TeNT was able to cleave syb II. In Figure 5C, SV2A/B KO neurons were largely protected from TeNT and these neurons could be re-sensitized with the infection of SV2A or B lentivirus (which infects greater than 90% of the cells) [28], [30]. In addition, we cultured SV2B and SV2A/B KO spinal cord neurons and observed that while SV2B KO mice were still sensitive to the addition of TeNT, SV2A/B double KO spinal neurons were protected from TeNT; again the neurons were re-sensitized to the toxin after infection with SV2A virus (Figure 5D). Together, these data indicate that SV2A and SV2B mediate entry of TeNT.

SV2 has three putative N-linked glycosylation sites at amino acids 498, 548, and 573 [53], [54], [55], [56], [57]. It has been shown that BoNT/E requires the third N-linked glycosylation site of SV2 to enter neurons [30], so we investigated whether the glycosylation of SV2 was also critical for TeNT entry. To address this, each of the three SV2A N-linked glycosylation sites were mutated to generate individual site mutants and were expressed in hippocampal neurons using lentivirus. None of the individual glycosylation mutations affected TeNT entry (Figure 5E). We note that PNGase F treatment cannot access all the N-glycosylation sites of SV2 in cultured neurons and triple glycosylation mutants of SV2 do not target properly to SVs, so we are unable to completely rule out whether the glycosylation of multiple sites might play a role in toxin binding or entry ([30];data not shown). However, the experiments reported here clearly demonstrate that – in contrast to BoNT/E [30] - loss of glycosylation at each of the individual glycosylation sites does not impact the entry of TeNT.

Next, we turned to an *in vivo* mouse model to investigate whether SV2B KO mice are resistant to TeNT intoxication. We injected WT and SV2B KO littermates with 5 µg/mouse of TeNT and determined the length of time required for the mice to expire. WT mice survived ∼190 minutes post-injection, while SV2B KO mice were resistant to TeNT and survived ∼400 minutes post-injection. The average survival time of KO mice (∼400 minutes) injected with 5 µg TeNT was longer than that of WT mice injected with 1 µg of TeNT (∼300 minutes) indicating the effective concentration of TeNT was reduced by at least five-fold in SV2B KO mice. (Figure 5F).

In order to determine whether the uptake of other toxins was altered in SV2A/B double KO neurons, we used BoNT/F, which also utilizes recycling SVs [58], as a control. We titrated BoNT/F from 0.3 to 10 nM on WT and knockout neurons and observed no significant difference in binding and entry, as evidenced by cleavage of syb II, between these two conditions (Figure 5G). These data indicate that loss of SV2 does not affect normal uptake of toxins that target SVs and furthermore, in contrast to previous suggestions, SV2A/B is not required for normal uptake of BoNT/F [50], [58].

### SV2A/B expression does not determine the targeting of TeNT to inhibitory spinal cord neurons

**Figure 6:**
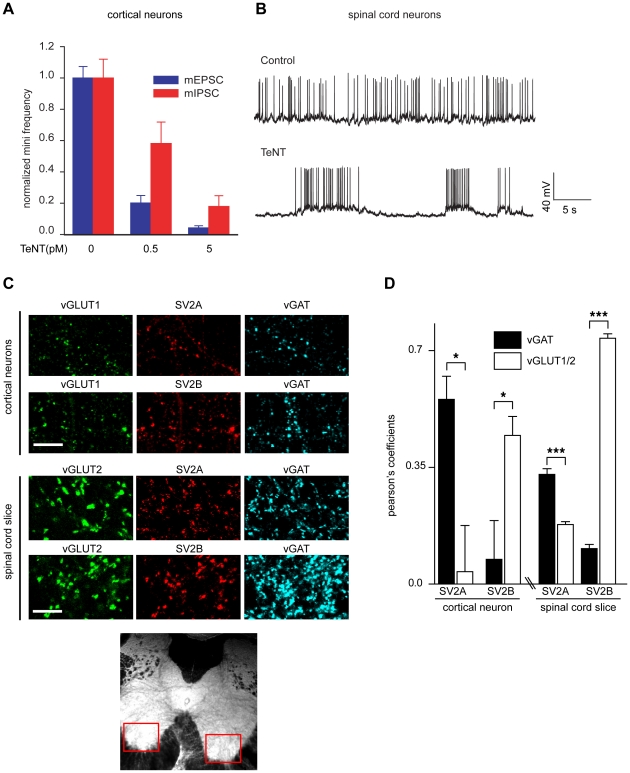
SV2A is largely expressed in inhibitory neurons while SV2B is predominately expressed in excitatory neurons. (A) Cultured cortical neurons were exposed to the indicated concentrations of TeNT for 3 minutes in high K^+^ buffer then returned to the incubator for 2 days. mEPSCs and mIPSCs were analyzed and the frequency of mEPSCs was reduced to a greater degree than mIPSCs. mEPSC/mIPSC at 0 pM TeNT, n = 10; mEPSC at 0.5 pM TeNT, n = 10; mIPSC at 0.5 pM TeNT, n = 9; mEPSC/mIPSC at 5 pM TeNT, n = 8; error bars represent SEM. (B) Sample traces of dissociated spinal cord neurons exposed to TeNT (50 pM) in high K^+^ buffer, washed than analyzed after 3 hours. Cultures treated with TeNT exhibited pronounced hyper excitability, as evidenced by high-frequency bursting activity, as compared to control neurons. (C) Representative images of SV2A/B staining with markers for excitatory (vGLUT1/2) or inhibitory (vGAT) boutons in cortical neurons and spinal cord slices. Red boxes indicate the region that was subjected to analysis by immunocytochemistry. (D) Pearson's coefficient for SV2A/B localization with excitatory/inhibitory terminals. In cortical neurons, SV2A localizes to inhibitory terminals while SV2B localizes to excitatory terminals. In contrast, in spinal cord slices, SV2A showed a greater degree of colocalization with markers for inhibitory terminals than excitatory terminals, while SV2B largely localized to excitatory terminals. Error bars represent SEM, SV2A cortical neurons n = 4, SV2B cortical neurons n = 6, spinal cord slice n = 13, *p<0.05, ***p<0.001.

To further understand how TeNT targets inhibitory neurons when released from MNs in the spinal cord, we first tested cortical neurons at low concentrations of TeNT to determine which population of neurons TeNT would affect first. Surprisingly in Figure 6A, at 0.5 pM toxin, miniature excitatory postsynaptic currents (mEPSCs) were reduced to 20% of control as compared to 60% for miniature inhibitory postsynaptic currents (mIPSCs). This is counter-intuitive because during the normal course of tetanus pathology, TeNT affects inhibitory neurons rather than excitatory neurons [1]. However, when spinal cord neurons were treated with 50 pM TeNT, typical hyper excitability of the culture – due to preferential loss of inhibitory transmission - was evident 3 hours after treatment (Figure 6B). However, extended incubation resulted in the inhibition of all action potentials (data not shown). Since we observed distinct effects in cortical and spinal cord neurons, we wondered whether different expression patterns of SV2 isoforms could be responsible for the opposite phenotypes observed in the two types of cultures. We found that among cortical neurons, SV2A is largely colocalized with inhibitory neurons, while SV2B is colocalized with excitatory neurons (Figure 6C–D). In adult spinal cord slices, we focused our studies on the ventral horn, where the cell bodies of MN are located. In this area, SV2A was more colocalized to inhibitory neurons and SV2B was largely colocalized with excitatory terminals. Since the SV2 expression patterns for spinal cord versus cortical neurons are similar, but TeNT has differential effects on excitatory versus inhibitory synaptic transmission in these two preparations, other yet to be defined factors must underlie the selective action of TeNT on inhibitory synaptic transmission observed *in vivo*.

## Discussion

Functionally, TeNT causes rigid paralysis, through a reduction of the strength of inhibitory inputs on MNs; however, the pathway through which TeNT enters neurons is unresolved. The majority of studies indicate that TeNT enters neurons through a non-SV pathway [35], [36], although one report concluded that the toxin enters via recycling SVs [34]. Among the CNTs, TeNT has the most unique intoxication pathway, yet little is understood the precise mechanism by which it reaches its target. In the current study, we demonstrate that in cultured hippocampal and spinal cord neurons, a receptor-binding fragment of TeNT exhibits robust binding to nerve terminals only after we stimulated robust SV exocytosis to deposit SV proteins into the plasma membrane. This result indicates that SVs harbor a receptor for the TeNT. We then extended these observations by determining whether full-length TeNT achieves functional entry by being internalized via recycling SVs. Indeed, in hippocampal neurons, as well as in inhibitory spinal cord neurons that are the physiological targets of TeNT, we observed markedly enhanced cleavage of TeNT's substrate, syb II, under conditions that stimulate SV exo- and endocytosis. In addition, dominant negative dynamin was also effective at inhibiting TeNT entry into hippocampal neurons. Finally, to inhibit SV exocytosis, spinal cord neurons or hippocampal neurons were pretreated with BoNT/A or E, respectively, and were protected from the effects of TeNT. Collectively, these findings firmly establish that the predominant pathway by which TeNT enters central neurons is through recycling SVs.

Most of the early efforts to identify the receptor for TeNT were focused on MNs where it was suggested that the TeNT receptor contained a PI-PLC sensitive GPI-anchor and was localized to lipid rafts [14], [35], [37]. This GPI-anchored protein was postulated to be the receptor important for retrograde transport of TeNT from the periphery of the MN to the soma, which is located in the spinal cord. Much of the literature has been dedicated to studying this non-SV receptor, but surprisingly, little work has been done to investigate the receptor in spinal cord neurons in the central nervous system. We provide the first direct evidence that a distinct vesicular compartment was required for entry into spinal cord neurons versus MNs [14], [35], [37], [59], [60]. Because neuronal activity and SV recycling were required for the cleavage of syb II, indicating that the toxin receptor resided on SVs, we began to screen SV proteins for TeNT binding activity. In order to achieve this, we used biotinylated toxins incubated with detergent solubilized brain extracts and identified the primary protein associated with TeNT to be SV2. To further confirm that TeNT uses SV2 as a receptor, we assayed for competition between TeNT and a toxin known to use SV2A/B as its receptor, BoNT/E [30]. Notably, we found that an excess amount of TeNT efficiently occluded the binding and entry of BoNT/E, consistent with competitive binding for sites on SV2.

To determine, definitively, whether TeNT relies on SV2 to bind and enter neurons, we took advantage of SV2A/B KO mice [51], [52]. We first cultured spinal cord neurons that lacked SV2A/B and monitored binding of a receptor-binding fragment of TeNT; WT neurons served as controls. It was observed that SV2B KO neurons had reduced binding and SV2A/B double KO neurons exhibited a further decrease in binding of HCR/T. Thus, both SV2A and B are important for TeNT to recognize and associate with the surface of central neurons. Furthermore, double KO neurons were largely protected from TeNT, as evidenced by the lack of cleavage of syb II; moreover, these neurons could be re-sensitized through infection with viruses that expressed SV2A or B. Using viruses that encode mutant forms of SV2, we found that glycosylation at any one of the three N-linked glycosylation sites was not required for TeNT to bind and enter neurons. In contrast, the third glycosylation site plays a critical role for binding and entry of BoNT/E and glycosylation appears to enhance the ability of BoNT/A to enter neurons via SV2 [30]. Given this result, in conjunction with the competition studies described above, TeNT and BoNT/E are unlikely to bind to the exact same sites on SV2, since BoNT/E requires glycosylation of the third N-linked glycosylation site in intra-lumenal loop 4 whereas TeNT does not. However, it seems likely that these two toxins compete for binding (Figure 4B–C) due to a steric hindrance; two different toxin molecules are unlikely to be able to simultaneously bind to the relatively small intra-lumenal loops of SV2 that are exposed to the extracellular milieu during exocytosis. Finally, intravenous injections revealed that SV2B KO mice are at least five-times more resistant to TeNT as compared to WT littermates.

The finding that SV2A/B double KO neurons exhibit decreased binding and entry of TeNT, in conjunction with the ability of TeNT to compete with BoNT/E, strongly implicates SV2 as the receptor for TeNT. However, it remains possible that loss of SV2 might prevent the proper expression or targeting of yet-to-be identified protein receptors for TeNT. Our experiments showing normal entry of BoNT/F, another toxin that requires SV recycling for entry [58], into SV2A/B double KO neurons argues against the notion that SV2 plays a general permissive role in toxin entry.

Interestingly, when we exposed cortical neurons to low concentrations of TeNT we discovered an unexpected preferential action on excitatory versus inhibitory neurotransmission. In contrast, in spinal cord cultures, TeNT preferentially acted on inhibitory neurons resulting in the expected pathological symptoms of hyper excitability. To further investigate how TeNT is directed to specific populations of neurons, we examined the distribution of SV2A and SV2B in both preparations. We found that SV2B expression is largely localized to excitatory terminals while SV2A is preferentially localized to inhibitory neurons in both cortical cultures and spinal cord slices; this differential distribution was more striking with cortical neurons. Thus, SV2A/B expression patterns do not seem to determine or dictate the specificity of TeNT for inhibitory neurons. It was previously suggested that structural features - especially the organization of neurons that is lost in dissociated culture - can be important for the selective action of TeNT on inhibitory neurons in the spinal cord [1]. Since inhibitory neurons typically synapse directly onto the cell body, while excitatory neurons form synapses on dendritic spines, it is possible that the retrograde carrier for TeNT predominately undergoes transcytosis at the cell body, thus allowing TeNT to preferentially target inhibitory neurons.

The retrograde transport of TeNT along axons of MNs, followed by transcytosis and final entry - through SVs - into inhibitory neurons is reminiscent of the transport and transcytosis of BoNT/A through the epithelial lining of the intestine and its subsequent selective entry into MNs. As TeNT and the BoNTs are related members of the CNT family, it is perhaps not surprising that these two toxins would both make use of transcytosis pathways, but that these pathways diverged during evolution to allow for distinct points of entry. TeNT is typically introduced through deep wounds, so it utilizes the MN as a mechanism of transport back to the spinal cord, where its enzymatic activity is focused on inhibitory neurons. BoNTs are typically ingested and need to enter the bloodstream to access the NMJ; this is achieved via transport across the epithelial layer in the gastrointestinal tract.

It is important to note that MNs also express SV2 [27]. This raises the question as to how TeNT selectively targets inhibitory interneurons, without affecting MNs; that is, why is the toxin not taken up into SV2-harboring SVs that acidify in MNs, thereby triggering translocation and resulting in flaccid paralysis (note: at extremely high concentrations, TeNT can in fact inhibit neurotransmitter release from MNs, probably via interactions with SV2 [61]). As alluded to above, previous studies indicated that SVs are not the major mode of entry as TeNT in MNs; e.g. TeNT shows little colocalization with the SV protein, syb II [59]. Another issue raised by the results presented here is the question of how TeNT is released from its receptor in MNs such that it can bind to SV2 on inhibitory interneurons following transcytosis.

In our first model, we envision that once TeNT reaches the NMJ, the protein receptor(s) responsible for retrograde transport (peripheral receptor) has a lower affinity for TeNT as compared to SV2. To prevent the entry of TeNT into SVs, the peripheral receptor would have to be present in large excess as compared to SV2, resulting in the sequestration of TeNT into the retrograde pathway. Next, TeNT undergoes retrograde transport back to the soma of the MN and the lack of acidification during transport prevents toxin translocation; hence the toxin does not gain access to the cytosol to cleave syb II [16]. During transcytosis, the peripheral receptor is no longer in abundance as compared to SV2. In addition, the higher affinity of SV2 for TeNT would allow for efficient capture of the toxin once it has dissociated from the MN, thus favoring binding and entry into the upstream inhibitory neuron.

Alternatively, recent data suggesting that PSGs are not internalized along with TeNT in MNs provide a second hypothesis [59]. As opposed to the previous model, the peripheral receptor, rather than SV2, has a higher affinity for TeNT. Therefore, once TeNT reaches a MN, it targets the peripheral receptor rather than SV2. As mentioned above, PSGs are important co-receptors for the CNTs. Once bound to PSGs and the peripheral receptor, TeNT is subsequently internalized into a vesicle bound for retrograde transport; however, PSGs are not internalized and remain on the plasma membrane [59], [62]. The loss of PSGs might dramatically reduce the affinity of TeNT for the peripheral receptor, relative to SV2, so TeNT can be released from the MN and target inhibitory neurons after transcytosis. Restated: the lack of internalization of PSGs into retrograde carriers might permit the release of the toxin from the MN. Further studies to identify the peripheral receptor will help address these questions.

In conclusion, we demonstrate that the recycling SVs are the primary mode of entry for TeNT into hippocampal and spinal cord neurons. Furthermore, SV2 is critical for the binding and entry of TeNT into neurons. To our knowledge, this is the first definitive identification of a protein receptor that is critical for the entry of TeNT into central neurons. This discovery identifies a new target that can be exploited to prevent tetanus. In addition, our greater understanding of the mechanism of TeNT entry should facilitate the development of a new class of therapeutics that allow for the delivery of drugs and genes to the central nervous system.

## Materials and Methods

### Ethics statement

All animal care and experiment protocols in this study were conducted under the guidelines set by the NIH *Guide for the Care and Use of Laboratory Animals* handbook. The protocols were reviewed and approved by the Animal Care and Use Committee (ACUC) at the University of Wisconsin - Madison (assurance number: A3368-01).

### Antibodies, materials, and mouse lines

Monoclonal antibodies directed against syb II (Cl. 69.1), SV2 (pan-SV2), syp (Cl. 7.2), and SNAP-25 (Cl. 71.1) were generously provided by R. Jahn (Max-Planck-Institute for Biophysical Chemistry, Gottingen, Germany). Rabbit polyclonal antibodies against BoNT/B and BoNT/E were described previously [29]. Guinea pig anti-vesicular glutamate transporter 1 and 2 (vGLUT1/2) antibodies were purchased from Chemicon (Temecula, CA). Mouse anti-FLAG antibody was purchased from Sigma-Aldrich (St. Louis, MO). Rabbit and mouse anti-vesicular GABA transporter (vGAT) and rabbit anti-SV2A and B antibodies were purchased from Synaptic Systems (Gottingen, Germany). Rabbit anti-HA tag and mouse anti-actin antibodies were purchased from Abcam (Cambridge, MA).

TeNT was purchased from List Biological Laboratories (Campbell, CA). BoNT/B and BoNT/E were purified as previously described [63], [64]. Tetrodotoxin was purchased from Sigma-Aldrich (St. Louis, MO). HCR/T, purified as previously described [65], was generously provided by J. Barbieri (Medical College of Wisconsin, Milwaukee, WI). SV2A, SV2B, and SV2A/B knockout mouse lines were previously described [52].

### Cell culture and spinal cord sections

Rat and mouse hippocampal neurons were cultured as described previously [30]. Cultured rat spinal cord neurons were prepared from embryonic (E) 14–15 day pups. SV2 knockout spinal cord neurons were prepared from E12.5∼14 timed pregnant mice. Spinal cord neurons were dissected in Hybernate E medium (Brain Bits, Springfield, IL), Spinal cords were cut into 12 pieces, incubated with 0.025% trypsin (Invitrogen, Carlsbad, California) for 15 minutes at 37°C, dissociated with DNase (20 µg/ml), washed with DMEM supplemented with 10% fetal bovine serum (FBS), and then triturated. Neurons were plated on 12 mm glass coverslips coated with poly-D-lysine and rat-tail collagen. Neurons were grown in DMEM with 10% FBS overnight. Afterwards, the media was replaced with Neurobasal medium supplemented with B-27 (2%) and Glutamax (2 mM). Neurons were used between 14–24 days in vitro (DIV). Transient transfection of neurons and lentiviral infections were performed as described previously [30]. Spinal cords were dissected from adult mice (4–6 months) and embedded in agarose. 300 µm serial sections were taken from the lumbar and thoracic sections with a vibratome.

### Immunocytochemistry and western blots

Neurons were incubated in the following buffers: TTX (150 mM NaCl, 4 mM KCl, 4 mM MgCl_2_, 10 mM D-glucose, 10 mM HEPES, 1 µM tetrodotoxin) and high K^+^ (same as TTX buffer but adjusted to 55 mM KCl, 99 mM NaCl, 2 mM CaCl_2_, 2 mM MgCl_2_ and without the addition of tetrodotoxin) at pH 7.4 with an osmolarity adjusted to 310 mOsm. Unless otherwise noted, neurons were incubated with toxins in high K^+^ buffer for 5 min. Images were collected with an Olympus FV1000 confocal microscope under a 60× water immersion lens (Melville, NY), Neuronal lysates were collected with 100 µl lysis buffer (20 mM Tris, 150 mM NaCl, 1% Triton X-100, 0.05% SDS, 0.5% PMSF, 0.5 µg/ml leupeptin, 0.7 µg/ml pepstatin, 1 µg/ml aprotinin, pH 7.4) per well (24-well plate). Lysates were subjected to SDS-PAGE and immunoblot analysis.

### Biotinylation of toxins and avidin bead pulldowns

0.4 mg of BoNT/B, E, and TeNT were dialyzed overnight at 4°C against 0.1M Na-MES buffer (pH 6.0). BoNT/A, B and TeNT were incubated with 0.025 mg EDC and 0.067 mg EZ-link biotin PEO_4_-amine at RT for 2 hr (Thermo Fisher, Waltham, MA). The reaction mixture was then dialyzed against PBS (pH 7.4) overnight at 4°C. 2.5 µg of biotinylated toxin was bound to neutravidin beads (Thermo Fisher, Waltham, MA) that were pre-blocked with 2% BSA and 0.1% cold water fish gelatin (Sigma-Aldrich, St. Louis, MO). Beads were incubated with rat brain detergent extracts and 250 µg/ml mixed PSGs for 1 hr at 4°C. Bound material (20%) was subjected to SDS-PAGE and immunoblot analysis.

### Mouse time-to-death assays

SV2B WT and KO littermates were injected intravenously with 100 µl of the indicated amount of TeNT resuspended in GelPhos (30 mM sodium phosphate, 0.2% gelatin, pH 6.3, autoclaved). Four mice were used in each experimental condition. Mice that survived longer than 420 minutes were euthanized.

### Electrophysiology

Whole-cell recordings were performed using a MultiClamp 700B amplifier (Molecular Devices). The bath solution consists of (in mM) 128 NaCl, 30 glucose, 5 KCl, 5 CaCl_2_, 1 MgCl_2_, 25 HEPES; pH 7.3. For recording action potentials or mEPSCs, the pipette solution contained (in mM) 125 K-gluconate, 10 KCl, 5 EGTA, 10 Tris-phosphocreatine, 4 magnesium ATP, 0.5 sodium GTP, 10 HEPES, pH 7.3 (305 mOsm). For recording mIPSCs, the pipette solution contained (in mM) 147 CsCl_2_, 2 EGTA, 5 Tris-phosphocreatine, 2 magnesium ATP, 0.5 sodium GTP, 10 HEPES, pH 7.3 (305 mOsm). To isolate AMPA receptor-mediated mEPSCs, 0.5 µM TTX (sodium channel blocker, Tocris), 50 µM D-AP5 (NMDA receptor antagonist; Tocris) and 20 µM bicuculline (GABA_A_ receptor antagonist; Tocris) were added. To isolate GABA_A_ receptor-mediated mIPSCs, bicuculline was replaced with 20 µM CNQX (AMPA receptor antagonist, Tocris). Recordings of mEPSCs and mIPSCs were performed in voltage-clamp mode with membrane potential held at −70 mV. Recordings of action potentials were performed in current-clamp mode with current held at 0 pA. Data were acquired using pClamp (Molecular Devices) software, sampled at 10 kHz, and filtered at 2 kHz. Off-line data analysis was performed using Clampfit (Molecular Devices) or MiniAnalysis (Synaptosoft) software. All experiments were carried out at room temperature.

### Statistical methods

Statistical significance was evaluated by two-tailed unpaired Student's t-test: *p<0.05, **p<0.01, ***p<0.001.

## References

[1] Schiavo G, Matteoli M, Montecucco C (2000). Neurotoxins affecting neuroexocytosis.. Physiol Rev.

[2] Faber K (1890). Die Pathogenie des Tetanus.. Berl Klin Wochenschr.

[3] Tizzoni G, Cattani G (1890). Untersuchungen uber das Tetanusgift.. Zentralbl Bakt.

[4] Montecucco C (1995). Clostridial neurotoxins : the molecular pathogenesis of tetanus and botulism.

[5] Schiavo G, Rossetto O, Santucci A, DasGupta BR, Montecucco C (1992). Botulinum neurotoxins are zinc proteins.. J Biol Chem.

[6] Blasi J, Chapman ER, Link E, Binz T, Yamasaki S (1993). Botulinum neurotoxin A selectively cleaves the synaptic protein SNAP-25.. Nature.

[7] Blasi J, Chapman ER, Yamasaki S, Binz T, Niemann H (1993). Botulinum neurotoxin C1 blocks neurotransmitter release by means of cleaving HPC-1/syntaxin.. Embo J.

[8] Schiavo G, Malizio C, Trimble WS, Polverino de Laureto P, Milan G (1994). Botulinum G neurotoxin cleaves VAMP/synaptobrevin at a single Ala-Ala peptide bond.. J Biol Chem.

[9] Schiavo G, Santucci A, Dasgupta BR, Mehta PP, Jontes J (1993). Botulinum neurotoxins serotypes A and E cleave SNAP-25 at distinct COOH-terminal peptide bonds.. FEBS Lett.

[10] Schiavo G, Benfenati F, Poulain B, Rossetto O, Polverino de Laureto P (1992). Tetanus and botulinum-B neurotoxins block neurotransmitter release by proteolytic cleavage of synaptobrevin.. Nature.

[11] Sudhof TC, Rothman JE (2009). Membrane fusion: grappling with SNARE and SM proteins.. Science.

[12] Chapman ER (2002). Synaptotagmin: a Ca(2+) sensor that triggers exocytosis?. Nat Rev Mol Cell Biol.

[13] Smith LDS, Sugiyama H (1988). Botulism : the organism, its toxins, the disease.

[14] Herreros J, Ng T, Schiavo G (2001). Lipid rafts act as specialized domains for tetanus toxin binding and internalization into neurons.. Mol Biol Cell.

[15] Lalli G, Bohnert S, Deinhardt K, Verastegui C, Schiavo G (2003). The journey of tetanus and botulinum neurotoxins in neurons.. Trends Microbiol.

[16] Bohnert S, Schiavo G (2005). Tetanus toxin is transported in a novel neuronal compartment characterized by a specialized pH regulation.. J Biol Chem.

[17] Deinhardt K, Salinas S, Verastegui C, Watson R, Worth D (2006). Rab5 and Rab7 Control Endocytic Sorting along the Axonal Retrograde Transport Pathway.. Neuron.

[18] Schwab ME, Suda K, Thoenen H (1979). Selective retrograde transsynaptic transfer of a protein, tetanus toxin, subsequent to its retrograde axonal transport.. J Cell Biol.

[19] Curtis DR, De Groat WC (1968). Tetanus toxin and spinal inhibition.. Brain Res.

[20] Montecucco C (1986). How do tetanus and botulinum toxins bind to neuronal membranes?. Trends in Biochemical Sciences.

[21] Kitamura M, Takamiya K, Aizawa S, Furukawa K, Furukawa K (1999). Gangliosides are the binding substances in neural cells for tetanus and botulinum toxins in mice.. Biochim Biophys Acta.

[22] Kitamura M, Igimi S, Furukawa K, Furukawa K (2005). Different response of the knockout mice lacking b-series gangliosides against botulinum and tetanus toxins.. Biochim Biophys Acta.

[23] Pierce EJ, Davison MD, Parton RG, Habig WH, Critchley DR (1986). Characterization of tetanus toxin binding to rat brain membranes. Evidence for a high-affinity proteinase-sensitive receptor.. Biochem J.

[24] Parton RG, Ockleford CD, Critchley DR (1988). Tetanus toxin binding to mouse spinal cord cells: an evaluation of the role of gangliosides in toxin internalization.. Brain Res.

[25] Yavin E, Nathan A (1986). Tetanus toxin receptors on nerve cells contain a trypsin-sensitive component.. Eur J Biochem.

[26] Deinhardt K, Schiavo G (2005). Endocytosis and retrograde axonal traffic in motor neurons.. Biochem Soc Symp.

[27] Dong M, Yeh F, Tepp WH, Dean C, Johnson EA (2006). SV2 is the protein receptor for botulinum neurotoxin A.. Science.

[28] Dong M, Tepp WH, Liu H, Johnson EA, Chapman ER (2007). Mechanism of botulinum neurotoxin B and G entry into hippocampal neurons.. J Cell Biol.

[29] Dong M, Richards DA, Goodnough MC, Tepp WH, Johnson EA (2003). Synaptotagmins I and II mediate entry of botulinum neurotoxin B into cells.. J Cell Biol.

[30] Dong M, Liu H, Tepp WH, Johnson EA, Janz R (2008). Glycosylated SV2A and SV2B mediate the entry of botulinum neurotoxin E into neurons.. Mol Biol Cell.

[31] Nishiki T, Kamata Y, Nemoto Y, Omori A, Ito T (1994). Identification of protein receptor for Clostridium botulinum type B neurotoxin in rat brain synaptosomes.. J Biol Chem.

[32] Mahrhold S, Rummel A, Bigalke H, Davletov B, Binz T (2006). The synaptic vesicle protein 2C mediates the uptake of botulinum neurotoxin A into phrenic nerves.. FEBS Lett.

[33] Rummel A, Karnath T, Henke T, Bigalke H, Binz T (2004). Synaptotagmins I and II act as nerve cell receptors for botulinum neurotoxin G.. J Biol Chem.

[34] Matteoli M, Verderio C, Rossetto O, Iezzi N, Coco S (1996). Synaptic vesicle endocytosis mediates the entry of tetanus neurotoxin into hippocampal neurons.. Proc Natl Acad Sci U S A.

[35] Munro P, Kojima H, Dupont JL, Bossu JL, Poulain B (2001). High sensitivity of mouse neuronal cells to tetanus toxin requires a GPI-anchored protein.. Biochem Biophys Res Commun.

[36] Parton RG, Ockleford CD, Critchley DR (1987). A study of the mechanism of internalisation of tetanus toxin by primary mouse spinal cord cultures.. J Neurochem.

[37] Herreros J, Lalli G, Montecucco C, Schiavo G (2000). Tetanus toxin fragment C binds to a protein present in neuronal cell lines and motoneurons.. J Neurochem.

[38] Greene CE (2006). Infectious diseases of the dog and cat.

[39] Schumaker HB, Lamont A, Firor WM (1939). The reaction of “tetanus sensitive” and “tetanus resistant” animals to the injection of tetanal toxin into the spinal cord.. J Immunol.

[40] Box M, Parks DA, Knight A, Hale C, Fishman PS (2003). A multi-domain protein system based on the HC fragment of tetanus toxin for targeting DNA to neuronal cells.. J Drug Target.

[41] Takamori S, Holt M, Stenius K, Lemke EA, Gronborg M (2006). Molecular anatomy of a trafficking organelle.. Cell.

[42] Lalli G, Herreros J, Osborne SL, Montecucco C, Rossetto O (1999). Functional characterisation of tetanus and botulinum neurotoxins binding domains.. J Cell Sci.

[43] Baldwin MR, Barbieri JT (2007). Association of botulinum neurotoxin serotypes a and B with synaptic vesicle protein complexes.. Biochemistry.

[44] Bizzini B, Stoeckel K, Schwab M (1977). An antigenic polypeptide fragment isolated from tetanus toxin: chemical characterization, binding to gangliosides and retrograde axonal transport in various neuron systems.. J Neurochem.

[45] Dumas M, Schwab ME, Baumann R, Thoenen H (1979). Retrograde transport of tetanus toxin through a chain of two neurons.. Brain Res.

[46] Morris NP, Consiglio E, Kohn LD, Habig WH, Hardegree MC (1980). Interaction of fragments B and C of tetanus toxin with neural and thyroid membranes and with gangliosides.. J Biol Chem.

[47] Weller U, Taylor CF, Habermann E (1986). Quantitative comparison between tetanus toxin, some fragments and toxoid for binding and axonal transport in the rat.. Toxicon.

[48] van der Bliek AM, Redelmeier TE, Damke H, Tisdale EJ, Meyerowitz EM (1993). Mutations in human dynamin block an intermediate stage in coated vesicle formation.. J Cell Biol.

[49] Simpson LL (1984). The binding fragment from tetanus toxin antagonizes the neuromuscular blocking actions of botulinum toxin.. J Pharmacol Exp Ther.

[50] Rummel A, Hafner K, Mahrhold S, Darashchonak N, Holt M (2009). Botulinum neurotoxins C, E and F bind gangliosides via a conserved binding site prior to stimulation-dependent uptake with botulinum neurotoxin F utilising the three isoforms of SV2 as second receptor.. J Neurochem.

[51] Crowder KM, Gunther JM, Jones TA, Hale BD, Zhang HZ (1999). Abnormal neurotransmission in mice lacking synaptic vesicle protein 2A (SV2A).. Proc Natl Acad Sci U S A.

[52] Janz R, Goda Y, Geppert M, Missler M, Sudhof TC (1999). SV2A and SV2B function as redundant Ca2+ regulators in neurotransmitter release.. Neuron.

[53] Scranton TW, Iwata M, Carlson SS (1993). The SV2 protein of synaptic vesicles is a keratan sulfate proteoglycan.. J Neurochem.

[54] Janz R, Sudhof TC (1999). SV2C is a synaptic vesicle protein with an unusually restricted localization: anatomy of a synaptic vesicle protein family.. Neuroscience.

[55] Feany MB, Lee S, Edwards RH, Buckley KM (1992). The synaptic vesicle protein SV2 is a novel type of transmembrane transporter.. Cell.

[56] Buckley K, Kelly RB (1985). Identification of a transmembrane glycoprotein specific for secretory vesicles of neural and endocrine cells.. J Cell Biol.

[57] Bajjalieh SM, Peterson K, Shinghal R, Scheller RH (1992). SV2, a brain synaptic vesicle protein homologous to bacterial transporters.. Science.

[58] Fu Z, Chen C, Barbieri JT, Kim JJ, Baldwin MR (2009). Glycosylated SV2 and gangliosides as dual receptors for botulinum neurotoxin serotype F.. Biochemistry.

[59] Deinhardt K, Berninghausen O, Willison HJ, Hopkins CR, Schiavo G (2006). Tetanus toxin is internalized by a sequential clathrin-dependent mechanism initiated within lipid microdomains and independent of epsin1.. J Cell Biol.

[60] Roux S, Colasante C, Saint Cloment C, Barbier J, Curie T (2005). Internalization of a GFP-tetanus toxin C-terminal fragment fusion protein at mature mouse neuromuscular junctions.. Mol Cell Neurosci.

[61] Matsuda M, Sugimoto N, Ozutsumi K, Hirai T (1982). Acute botulinum-like intoxication by tetanus neurotoxin in mice.. Biochem Biophys Res Commun.

[62] Nichols BJ (2003). GM1-containing lipid rafts are depleted within clathrin-coated pits.. Curr Biol.

[63] Evans DM, Williams RS, Shone CC, Hambleton P, Melling J (1986). Botulinum neurotoxin type B. Its purification, radioiodination and interaction with rat-brain synaptosomal membranes.. Eur J Biochem.

[64] Schmidt JJ, Siegel LS (1986). Purification of type E botulinum neurotoxin by high-performance ion exchange chromatography.. Anal Biochem.

[65] Chen C, Fu Z, Kim JJ, Barbieri JT, Baldwin MR (2009). Gangliosides as high affinity receptors for tetanus neurotoxin.. J Biol Chem.

